# Optimizing detectability of the endangered fan mussel using eDNA and ddPCR

**DOI:** 10.1002/ece3.10807

**Published:** 2024-01-28

**Authors:** Virginie Marques, Géraldine Loot, Simon Blanchet, Claude Miaud, Serge Planes, Claire Peyran, Véronique Arnal, Coralie Calvet, Sylvain Pioch, Stéphanie Manel

**Affiliations:** ^1^ CEFE, Univ Montpellier, CNRS, EPHE‐PSL University, IRD, Univ Paul Valéry Montpellier 3 Montpellier France; ^2^ Ecosystems and Landscape Evolution, Institute of Terrestrial Ecosystems, Department of Environmental System Science ETH Zürich Zürich Switzerland; ^3^ Land Change Science Swiss Federal Research Institute WSL Birmensdorf Switzerland; ^4^ EDB, Laboratoire Ecologie et Evolution (UMR 5174) Université de Toulouse, UPS, CNRS, IRD Toulouse France; ^5^ SETE, Station d'Écologie Théorique et Expérimentale (UAR2029), Centre National pour la Recherche Scientifique Moulis France; ^6^ PSL Research University: EPHE – UPVD – CNRS, UAR 3278 CRIOBE Perpignan France; ^7^ AgroParisTech Paris France; ^8^ Montpellier Research in Management (MRM) Univ Montpellier, Univ Paul Valéry Montpellier 3, Univ Perpignan Via Domitia Montpellier France; ^9^ CEFE, Univ Montpellier, CNRS, EPHE‐PSL University, IRD Montpellier France; ^10^ Institut Universitaire de France Paris France

**Keywords:** ddPCR, eDNA, Mollusk, monitoring, qPCR, reproduction

## Abstract

Spatial and temporal monitoring of species threatened with extinction is of critical importance for conservation and ecosystem management. In the Mediterranean coast, the fan mussel (*Pinna nobilis*) is listed as critically endangered after suffering from a mass mortality event since 2016, leading to 100% mortality in most marine populations. Conventional monitoring for this macroinvertebrate is done using scuba, which is challenging in dense meadows or with low visibility. Here we developed an environmental DNA assay targeting the fan mussel and assessed the influence of several environmental parameters on the species detectability in situ. We developed and tested an eDNA molecular marker and collected 48 water samples in two sites at the Thau lagoon (France) with distinct fan mussel density, depths and during two seasons (summer and autumn). Our marker can amplify fan mussel DNA but lacks specificity since it also amplifies a conspecific species (*Pinna rudis*). We successfully amplified fan mussel DNA from in situ samples with 46 positive samples (out of 48) using ddPCR, although the DNA concentrations measured were low over almost all samples. Deeper sampling depth slightly increased DNA concentrations, but no seasonal effect was found. We highlight a putative spawning event on a single summer day with much higher DNA concentration compared to all other samples. We present an eDNA molecular assay able to detect the endangered fan mussel and provide guidelines to optimize the sampling protocol to maximize detectability. Effective and non‐invasive monitoring tools for endangered species are promising to monitor remaining populations and have the potential of ecological restoration or habitat recolonization following a mass mortality event.

## Graphical abstract

The study developed an environmental DNA (eDNA) assay to detect the critically endangered fan mussel in the Mediterranean area. The eDNA marker used with ddPCR effectively identified the species in water samples, with deeper sampling depths showing slightly higher DNA concentrations. This eDNA approach holds promise for non‐invasive monitoring and potential restoration of endangered species populations.

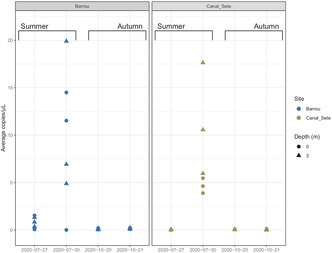

## 1 INTRODUCTION

Monitoring biodiversity is critical to assess the effects of rising anthropogenic stressors on natural ecosystems, as well as evaluating and implementing policy and management initiatives (Díaz et al., 2019). Spatial and temporal monitoring of species threatened with extinction is especially important to quickly detect declining populations and to set appropriate conservation measures (Robinson et al., 2018). Since many threatened species have national or international protected statutes, reliable monitoring, and detection are crucial for various purposes, such as species distribution modeling, landscape planning and the creation of potentially impacting infrastructures in their habitats to adopt measures to avoid or minimize their destruction (Loiseau et al., 2020; Woinarski et al., 2017). Efficient monitoring methods are needed for environmental managers and stakeholders to accurately detect protected or species of interest, while also minimizing sampling effort, economic costs, mortality, and disturbance to threatened species (Scheele et al., 2019).

On the Mediterranean coast, the fan mussel (*Pinna nobilis*) is critically endangered and under a protected species list both nationally and internationally (Annex IV of European Community, 1992; Annex II Barcelona convention 1976; Annex IV French habitat directive). It is endemic to the shallow waters of the Mediterranean region from Spain to Turkey, exploited in antiquity by the Romans for its shell and byssus. The fan mussel is the second largest invertebrate in the world with up to 1 m in height. Its reproduction season ranges from June to August, during which gametes are emitted by the successive hermaphrodite adults to be dispersed via sea currents (Degaulejac, 1995). In 2019, the fan mussel was listed as critically endangered (CR) in the IUCN red list (Kresting et al. 2019), following a mass mortality event. Over the entire basin, a disease outbreak caused by the protozoan parasite *Haplosporidium pinnae* that emerged in Spain in 2016 devastated fan mussel populations (Cabanellas‐Reboredo et al., 2019; Vázquez‐Luis et al., 2017). Since then, the disease has spread, and marine populations of *P. nobilis* have been decimated in France and Italy, where most sites showed almost 100% mortality (Cabanellas‐Reboredo et al., 2019; García‐March et al., 2020). A 800 km coastline survey in Italy in 2022 found no live individual remaining (Pensa et al., 2022). The last known environment harboring healthy fan mussels are brackish lagoons surrounding the Mediterranean shore (Nebot‐Colomer et al., 2022; Peyran et al., 2022).

Given the important threats to the fan mussel, it is critical to use accurate methods to detect and monitor its remnant populations while limiting disturbances. Such methods should be able to detect rare or elusive species that are difficult to detect using traditional methods (Mathon et al., 2022). Indeed, most biomonitoring for large benthic invertebrates including the fan mussel is done using scuba, so detectability is impaired when the water visibility is limited due to weather conditions or in murky waters such as estuaries, brackish waters, or harbors (Centoducati et al., 2007; Katsanevakis et al., 2021). Low abundance of the species further challenges its detection. The fan mussel is often associated with *Posidonia oceanica* seagrass meadows and buried up to 1/3 of its height in the sediment, which further challenge the visual detection of the smallest individuals, or even adults when meadows are dense (Richardson et al., 1999). Its status as a protected species by the European habitat directive (Council Directive 92/43/EEC of 21 May 1992) prohibits developers from destroying it. Environmental DNA (eDNA) analyses are now commonly used for both full community screening and species‐specific detection assays (Deiner et al., 2017) and used as a complementary or alternative method to more traditional destructive and/or visual‐based detection methods (Cole et al., 2021; Deiner et al., 2017; Polanco Fernández et al., 2020). eDNA relies on discarded tissue material in the environment from organisms, where DNA can then be extracted and PCR amplified. Species‐specific assays require the development of specific markers (Klymus et al., 2020), and are generally amplified using quantitative PCR (qPCR) or digital droplet PCR (ddPCR), either with or without probes to increase specificity (Brys et al., 2021). Some studies suggest that ddPCR is more sensitive than qPCR to detect rare species with low abundances in the environment (Dimond et al., 2022; Mauvisseau et al., 2019, but see Johnsen et al., 2020). Additional advantages of ddPCR include a better tolerance to PCR inhibitors present in plants, soil, water, and food (Morisset et al., 2013; Rački et al., 2014) and the access to DNA absolute quantification without relying on a standard curve (Hunter et al., 2018).

Many eDNA species‐specific assays have been developed for aquatic species from fish to invertebrates such as mussels or crayfish (Hernandez et al., 2020; Uthicke et al., 2018), but ‐up to our knowledge‐ no eDNA‐based assay exists to specifically target the fan mussel. Generally, despite their importance in aquatic systems, invertebrates tend to be underrepresented in eDNA studies (Belle et al., 2019). While eDNA methods are now increasingly used to monitor some freshwater mussel species (Prié et al., 2023; Stoeckle et al., 2021), there is still a lack of applicable approaches for marine mussels like the fan mussel. Invertebrates are known to shed less DNA compared to organisms such as fish, making their detection more challenging using DNA‐based methods (Andruszkiewicz Allan et al., 2021). Mauvisseau et al. (2019) failed to detect an endangered freshwater mussel using qPCRs, and detected low DNA concentration using ddPCRs. Some parameters are known to influence eDNA detection and quantification, such as the distance to the organisms (Murakami et al., 2019), water temperature (Lacoursière‐Roussel et al., 2016), water chemistry or turbidity (Stoeckle et al., 2017), or spawning events (Bracken et al., 2019; Bylemans et al., 2017), but quantifying the exact effect of each parameter on species detectability remains challenging. From an applied perspective, environmental managers require clear guidelines to design appropriate biomonitoring programs that maximize species detectability using DNA‐based methods.

This study aims at (i) developing and testing an eDNA assay to detect the fan mussel, (ii) providing a proof of concept on whether the fan mussel can be detected from environmental samples, and (iii) determining the influence of several environmental and sampling parameters on detectability and DNA concentration of the fan mussel in situ. To address these objectives, we designed and tested a mitochondrial marker targeting the fan mussel using in silico and in vitro testing. Then, we applied the ddPCR method from in situ samples in a Mediterranean lagoon with different densities of fan mussels previously estimated with visual‐based methods. We investigate the effects of density, sampling depth, and season on fan mussel eDNA detectability. We hypothesize an effect of abundance, depth, and season, with higher detectability in deeper samples as the fan mussel is a sessile benthic invertebrate, and in summer during the reproduction and higher metabolism activity season.

## 2 METHODS

### 2.1 Assay development

Reference sequences on the mitochondrial genome were downloaded for *P. nobilis* and co‐occurring related species of the same family (*Pinna rudis* and *Atrina fragilis*) from EMBL (Kanz et al., 2005), and aligned using Geneious Prime 2020 (https://www.geneious.com/). Primer selection was done by maximizing specificity on the binding sites for the target species while maximizing the number of mismatches of ligation sites of closely related species. Primers were designed manually with the assistance of the primer3 algorithm on Geneious and amplified a sequence insert of ~202 bp on the mitochondrial COI gene for *P. nobilis* (Table S1), the full amplified sequence being ~243 pb. The selected primer pair (PN_COI_M15; forward‐TCAGCTTTTGTAGAGGGCGG; reverse‐ AGAGACTACCAACAGCACAGC) was also tested on the entire NCBI database using in silico PCR with the ecoPCR software (Boyer et al., 2016) allowing up to three mismatches on each primer (so six in total), to verify the absence of unrelated species cross‐amplification. Additionally, a probe (PN_COIM15‐Probe; FAM‐5′ TGGATTTGTTCCCTTGGGCTGTTC 3′‐ BHQ1) was designed to enhance specificity using the Primer3Plus software (Untergasser et al., 2012)

We tested this marker on *Pinna nobilis* tissues (nine individuals from several French coastal localities) and both *A. fragilis* and *P. rudis* (five and three individuals, respectively) (Table S2) all preserved in 96% ethanol. Amplification tests were done both with the primers only, and with the combination of the primer and the probe. DNA was extracted using the Blood and Tissues Qiagen kit following manufacturer's instructions. Reactions were performed with 0.5, 1, or 2 μL of template DNA extract, 5‐μL ReadyMix (at 2×; REDExtract‐N‐Amp PCR readyMix, Sigma‐Aldrich), and 2‐μL of each primer (at 2 pM). Thermocycling parameters were: 95°C for 30 s, 40 cycles of 95°C for 30 s. 60°C for 30 s. 72°C for 1 min, and a final elongation step of 72°C for 5 min. Purification and Sanger sequencing of PCR products were carried out by Eurofins Genomics (Ebersberg. Germany). Electropherograms were checked using Geneious Prime 2020 (https://www.geneious.com).

### 2.2 Sampling for eDNA

DNA sampling aimed to first collect live fan mussel from an aquarium and then sample water in real field condition in a Mediterranean lagoon with known populations of fan mussel.

We took two 10 L‐samples of water from an aquarium containing live fan mussels with a density of four mussels in 60 L at the Biodiversarium aquarium in Banyuls‐sur‐Mer (France, year 2020) to first test our assay on a controlled environment. Water sampling was performed using an Athena peristaltic pump (Proactive Environmental Products, Bradenton, Florida) with a 1.0 L/min flow. Water was filtered through a VigiDNA 0.20 μm cross‐flow filtration capsule (SPYGEN, le Bourget du Lac, France) and immediately after filtration, each filter unit was filled with CL1 Conservation buffer (SPYGEN) and stored at room temperature (20–25°C) until DNA extraction.

**FIGURE 1:**
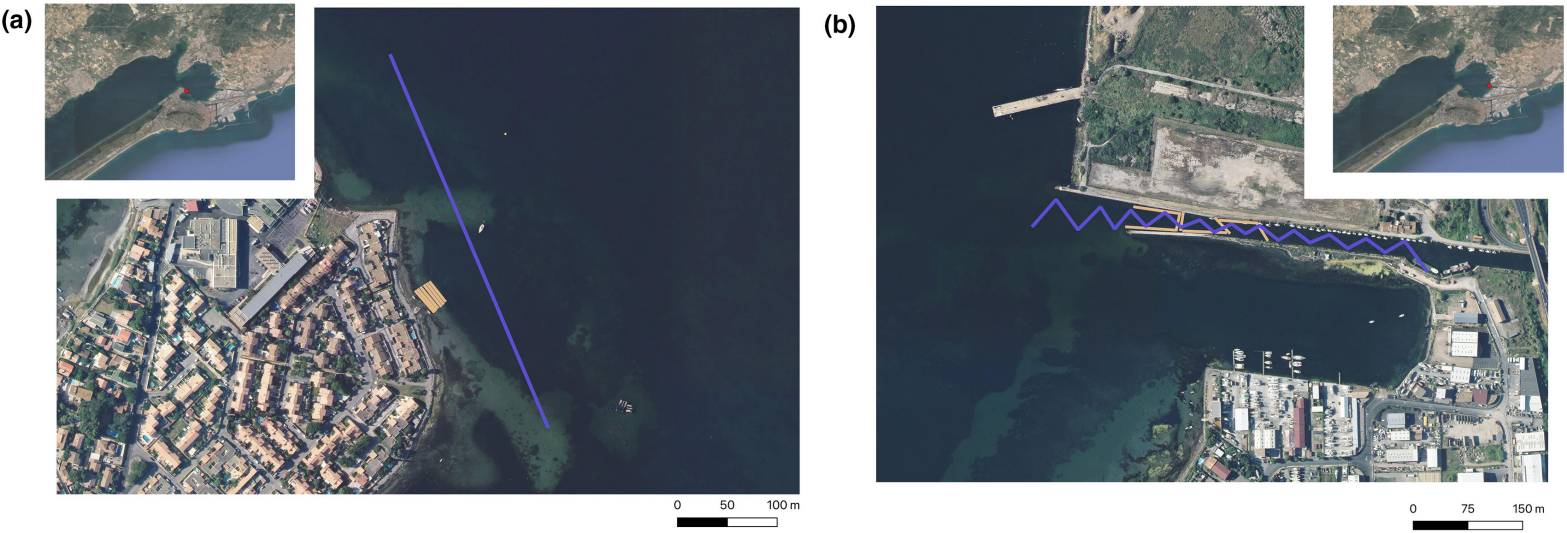
Sampling strategy on the Thau lagoon with (a) the Barrou site and (b) the Canal de Sète site. Purple lines indicate the transects done with the boat, orange lines indicate the dive transects done in Foulquie et al., 2020, to infer fan mussel density locally.

In a second step, we sampled water in the field with known presence and densities of fan mussels in the Thau lagoon (Sète, France) (Foulquie et al., 2020), one of the last known locations to harbor healthy mussel populations in France. Sampling sites were chosen from the study of Foulquie et al. (2020) which assessed fan mussel densities in several sites around the lagoon 3 months prior to our sampling. We selected sites with at least 3 m of depth and varying densities: the Barrou (~9 ind/100 m^2^) and the Sete Canal (~4 ind/100 m^2^) (Figure 1). Maximum depth of those sites was ~2.5 m. We filtered water from a boat using the same pump and settings as for the aquaria samples but made linear transects of ~300 m over the site area, for a total of 30 L per sample. Transects were made at low speed (5 knots) and by going back and forth to remain in the area, with one pump on each side of the boat and using disposable tubing and gloves. Surface samples were done at ~0.5 m of the surface with short tubes, and deeper samples were done to target the benthos using 3 m‐long weighted tubes. We chose to sample seasonally during Summer and Autumn to encompass various environmental conditions and test a potential effect of the reproduction period, known to occur during the summer months. We collected a total of 48 samples, with 24 samples over 2 days in summer in July (July 27, 2020 and July 30, 2020), and 24 samples over 2 days in autumn in October (October 20, 2020 and October 21, 2020). Each day, 12 samples were collected spanning both sites and two sampling depths (bottom and surface), so that three replicates were obtained for each site‐depth combination.

### 2.3 eDNA extraction and amplification by qPCR and ddPCR

#### 2.3.1 DNA extraction

DNA extraction was performed at SPYGEN (Le Bourget du Lac, France) following the protocol described in Polanco Fernández et al. (2020), in a dedicated laboratory for eDNA extraction with UV treatment and positive air pressure. Briefly, each capsule was agitated for 15 min on an S50 shaker (cat Ingenieurbüro™) at 800 rpm. The buffer was then emptied into two 50‐mL tubes before being centrifuged for 15 min at 15,000 *g*. The supernatant was removed with a sterile pipette, leaving 15 mL of liquid at the bottom of each tube. Then, 33 mL of ethanol and 1.5 mL of 3 M sodium acetate were added to each 50‐mL tube and stored for at least one night at −20°C. The tubes were centrifuged at 15,000 *g* for 15 min at 6°C, and the supernatants were discarded. After this step, 720 μL of ATL buffer from Qiagen Blood and Tissue Kit (Qiagen GmbH) was added to each tube. Each tube was then vortexed, and the supernatant was transferred to a 2‐mL tube containing 20 μL of Proteinase K. The tubes were finally incubated at 56°C for 2 h. Subsequently, DNA extraction was performed using NucleoSpin® Soil (MACHEREY‐NAGEL GmbH & Co.) starting from step 6 and following the manufacturer's instructions, and two DNA extractions were carried out per filtration capsule. The elution was performed by adding 100 μL of SE buffer twice. The two DNA samples were pooled before the amplification step. After the DNA extraction, the samples were tested for inhibition by qPCR (Biggs et al., 2015). If the sample was considered inhibited, it was diluted fivefold before amplification.

#### 2.3.2 Amplification with ddPCR

ddPCRs were run with a Bio‐Rad QX200 Droplet Digital PCR system™ (Bio‐Rad, Temse, Belgium). Each 22 μL ddPCR reaction mix contained 1× Bio‐Rad ddPCR supermix for probes (no dUTP), 900 nM forward primer, 900 nM reverse primer, 250 nM probe, 2,5 μL template, and 3,99 μL H2O. ddPCR reaction was placed in a QX200 Droplet Generator to generate approximately 20,000 droplets in which independent PCR reactions occur. PCR was performed with the following thermal conditions: 95°C for 10 min followed by 40 cycles of 95°C for 30 s and 58°C for 1 min; and 98°C for 10 min and 4°C for 30 min. Optimal annealing temperature (58°C) was determined based on an initial thermal gradient experiment testing temperatures from 54 to 64°C. Droplets were then read on a QX200 droplet reader (Bio‐Rad). Each run included three PCR positive and three PCR negative controls and samples were tested in triplicate (*N* = 3). QuantaSoft software was used to count the PCR positive and PCR negative droplets and to provide absolute quantification of target DNA. The baseline threshold for separating positive and negative droplets was manually chosen per run, based on the distribution of the negative droplets from the negative control wells. The quantification measurements of each target were expressed as the copies number per 1 μL of reaction.

### 2.4 Analysis

Amplification results from ddPCR were analyzed both considering the number of positive replicates among the three replicates per sample and quantitatively using the number of copies per μL. Repeatability by sample between the PCR replicates was assessed with the R package rptR (Stoffel et al., 2017). The average number of copies per μL measured with ddPCR were related to site, depth, and season using a general linear model (GLM). We used a Poisson distribution to model the average number of copies per μL among the three replicates for each sample. We added a dummy variable representing a putative reproduction event on a particular summer sampling day.

## 3 RESULTS

### 3.1 Barcode development

We tested the specificity of our designed primer pair (PN_COI_M15) using in silico PCR amplification on (i) our target species and (ii) closely related species occurring in the Mediterranean area. There were at least four mismatches on a single primer (forward or reverse) on closely related species, and no mismatches on our target species (Figure S1). In silico amplifications only take into account sequences long enough to contain the entire barcode, so we manually checked specificity on more sequences by also including shorter aligned sequences, so not containing the entire barcode or primer binding zones. We found that specificity remained unchanged for all but two sequences from Italy, where we found one mismatch close to the 5′ end of the reverse primer. In silico PCR on the entire EMBL database revealed that no other species were amplified with less than three mismatches on both forward and reverse primers (Figure S1). All nine *P. nobilis* tissues were amplified using our developed marker, while no tissus from *Atrina fragilis* were amplified (Table S2). However, all three individuals of *Pinna rudis* were amplified by our marker revealing the non‐specificity of the marker. All PCR products were sequenced to validate species assignments, and *Pinna rudis* amplifications were confirmed despite four mismatches on primer binding sites. We further tested the amplification of both *P. nobilis* and *P. rudis* using the ddPCR approach for which a probe (which theoretically increases specificity) has been designed (see above). All tissues (either diluted or not) from both *P. nobilis* and *P. rudis* were successfully amplified, confirming the poor specificity of the designed probes, despite the setting of the probe. Nonetheless, the fluorescence intensity was significantly lower for *P. rudis* (3154.37 ± 64.55, mean ± 95% CI) than for *P. nobilis* (4901.61 ± 270.38, mean ± 95% CI), demonstrating a higher specificity toward *P. nobilis* and a fluorescence threshold to infer the most likely species being amplified.

### 3.2 The ddPCR for aquarium samples

The aquaria samples containing only *P. nobilis* were accurately amplified by ddPCR with all three PCR replicates being positive with a mean of 301 copies/μL and with a mean fluorescence intensity of 4755.5 (±270.38, 95% CI), that is, within the range of fluorescence found for *P. nobilis* tissues.

### 3.3 The ddPCR approach for lagoon eDNA samples

**FIGURE 2:**
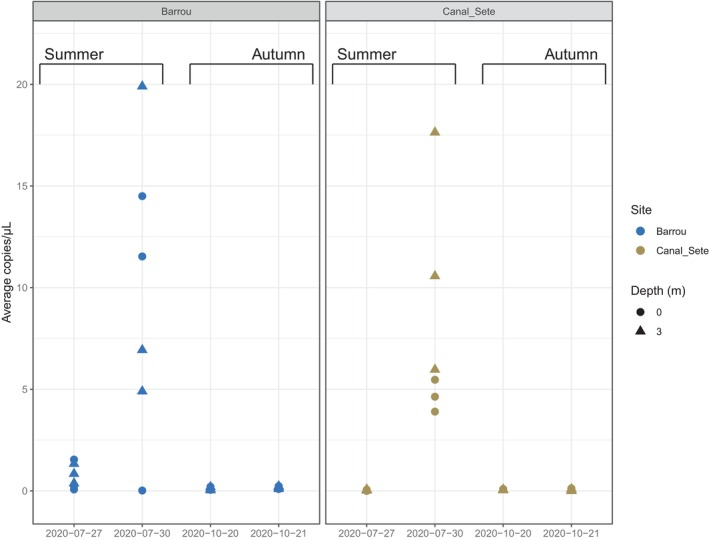
Average number of DNA copies (of three ddPCR replicates) depending on site, season, and depth.

Out of the 48 samples, 46 had at least one positive replicate (out of three) using the ddPCR assay, with measured copy number ranging from 0.05 to 20 copies/μL. The mean fluorescence of positive droplets was 5107.60 (±139.6, 95% CI), a range similar to that observed for positive controls (tissues and aquaria, see above) and largely higher than the range of fluorescence observed for tissues of *P. rudis* (see above). DNA quantification was highly consistent among the three replicates, with a repeatability of *R* = .88 (sd = 0.029, CI = [0.816, 0.925]). Only one replicate was necessary for detection, except for the lowest values with less than 0.15 copies/μL for which at least two replicates were needed to identify a positive sample (Figure S2). All samples measured with more than 5 copies/μL were collected the same day in summer (July 30, 2020) at both sites (Figure 2). Samples collected 3 days before (July 27, 2020) did not yield as much DNA, with concentrations similar to autumn values for one site (Sete_Canal, range 0–0.07 copies/μL) or slightly higher than autumn for the other site (Barrou, range 0.07–1.5 copies/μL) (Figure 2).

### 3.4 Parameters influencing eDNA concentration

**FIGURE 3:**
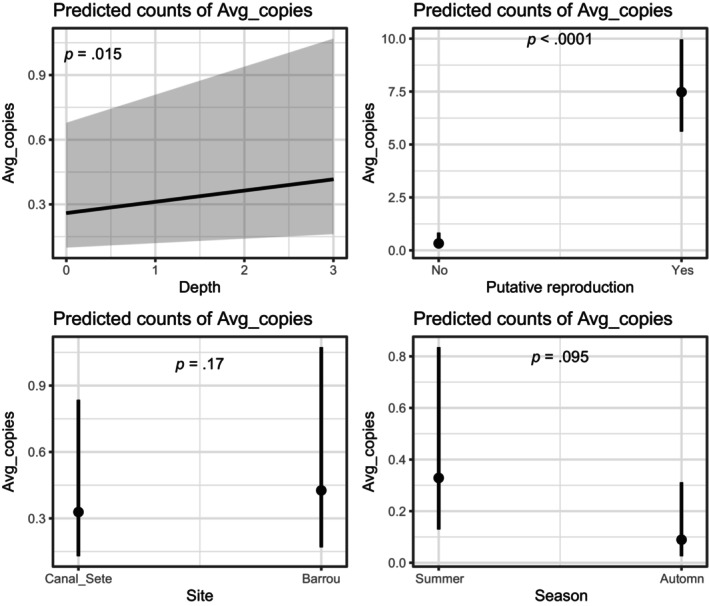
Parameter effects from the Poisson regression linking the ddPCR DNA copy number with different explanatory variables, considering a putative reproduction event on the July 30, 2020 sampling day.

Modeling the DNA concentrations measured by ddPCR with a GLM revealed a positive influence of sampling depth and a potential reproductive event, but no effect of season or species abundance. When excluding the dummy variable representing the putative reproduction event in the GLM, sampling depth and season both influenced DNA concentration with season having the strongest effect (season: estimate: 3.78, z‐value: 5.9, *p* < 10–9) (Table S3). Summer season and deeper samples (3 m) lead to higher DNA concentration than autumn season and shallower samples (0 m) (Figure S3). When adding the dummy variable for the putative reproduction event to make sure seasonal effect is not driven by a single day, the seasonal effect was no longer significant (estimate: 1.30, z‐value: 1.7, *p* = .095) (Table 1), whereas depth still had a significant effect on eDNA concentration (Table 1, Figure 3). For both models, sampling sites represented by distinct densities of fan mussels did not affect detectability nor DNA concentration.

**TABLE 1:** Outputs of the Poisson regression used to link the number of DNA copies measured by ddPCR with the different explanatory variables (depth, reproduction (putative), site, season).

	Estimate	Std. Error	Z value	Pr(>|z|)
(Intercept)	−2.39168	0.64517	−3.707	<.001
Depth	0.15736	0.06443	2.442	.015
Reproduction (putative)	3.1247	0.47348	6.599	<.001
SiteCanal_Sete	−0.26049	0.18962	−1.374	.17
SeasonSummer	1.30323	0.78103	1.669	.10

*Note*: Coefficient estimates, Z ‐tests values and related values are provided, including the dummy parameter of a putative reproduction even on the July 30, 2020 sampling day.

## 4 DISCUSSION

We successfully designed a molecular assay able to amplify the endangered fan mussel (*P. nobilis*) that was validated both in controlled and field conditions. The present assay was not specific enough as it also amplified a closely related species, *P. rudis*, and would require to be completed by a sequencing step to distinguish the two species. ddPCR was able to amplify DNA from environmental samples in almost all samples despite the low DNA concentration. Almost all samples had very low DNA concentration impairing the accurate detection of the species in an environmental management context. Our models indicate that season and species density did not influence eDNA concentration while increased sampling depth close to the seafloor and a suspected reproductive event could have enhanced eDNA concentration (Figure 3).

Designing specific molecular assays for PCR amplification is challenging (Hernandez et al., 2020; So et al., 2020; Thalinger et al., 2020). In this study, the marker we developed is unfortunately not fully specific to our target species as it also amplifies a closely related species, *P. rudis*, which is not endangered. *Pinna rudis* status has not been evaluated by the IUCN red list, but is still part of the Bern convention under the Annex II: strictly protected fauna species (https://eunis.eea.europa.eu/species/Pinna%20rudis). Challenges to design specific markers are common due to the genetic proximity of co‐occurring species, making the design of markers easier for non‐indigenous species outside their native range as there is generally no close species in the invaded range (Ardura et al., 2015). Identifying genetic regions with sufficient mismatches between *P. nobilis* and *P. rudis* was challenging as they show low mitochondrial genetic differentiation and also hybridize (Vázquez‐Luis et al., 2021). While both species occur in the Mediterranean Sea, *P. rudis* is mostly restricted to the warmest areas of western Mediterranean (mostly in Spain) and remains rare in the East Mediterranean (Gvozdenović et al., 2019). The only known location of *P. rudis* in France is in Corsica Island (Gvozdenović et al., 2019; Vicente, 2021). *Pinna rudis* is not known to be present in the Thau lagoon, thus we can confidently conclude that detection signals were associated with *P. nobilis*. Moreover, the fluorescence intensity measured from ddPCR in environmental DNA signals matched those from aquaria and tissues found from the pen shell, further strengthening the claim that *P. nobilis* was the species detected in our environmental samples. The PN_COI_M15 marker can accurately be used to detect *P. nobilis* in areas where *P. rudis* is known to be absent, whereas when both species co‐occur unspecific amplification could happen and may bias conclusions. To validate a specific detection, even in the absence of unspecific amplification, Thalinger et al. (2020) recommend to systematically sequence PCR products. Here, we would recommend a combination of fluorency measures from the ddPCR with PCR products sequencing when *P. rudis* is allegedly absent before validating the presence of *P. nobilis* in a given site. Beyond the proof of concept and for a management context, it would be necessary to design a new assay with sufficient specificity to the target species (or to the other species) to validate detections with a high probability. We designed two other candidate markers on the COI and 16S mitochondrial genes, but they were excluded as they showed a lighter PCR band on the electrophoresis gel in the initial screening tests, indicating a potential lower amplification power of fan mussel tissues. They, however, proved to be specific to *P. nobilis* as they did not amplify any closely related species (see Table S2 for the primer sequences)and are thus plausible alternatives to investigate. To counter the challenges to design and test species‐specific PCR primers for closely related species, CRISPR‐Cas approaches could be promising as there are multiple specificity filtering steps with initial PCR or RPA amplification followed by the use of a Cas enzyme on a single ~30 pb gene section (Williams et al., 2019).

Identifying parameters influencing the detectability and concentration of an eDNA signal is of critical importance when designing sampling or giving guidelines to environment managers (Goldberg et al., 2016). Among the important known parameters are the distance to the DNA source, temperature, biomass, and water stratification (Allan et al., 2021; Harrison et al., 2019; Jo et al., 2019; Rourke et al., 2022). In river systems, DNA is known to be transported as a fine particulate organic matter (FPOM) and can travel up to several kilometers downstream, depending on river width and water flow (Carraro et al., 2018; Pont et al., 2018; Shogren et al., 2017). In marine systems where water flow is more complex, much less is known regarding the behavior of eDNA particles, but theoretical models and experiments suggest a fastest dilution around the DNA source, with only a few meters to tens meters of detectable signal before falling below the detection threshold (Allan et al., 2021; Harrison et al., 2019; Murakami et al., 2019). This stresses the importance of sampling as close as possible to the DNA source in order to maximize DNA recovery. In a brackish lagoon, we showed a significant effect of sampling depth on DNA concentration even within a restricted range—that is, from the surface to the bottom at 3 m depth below the surface—with higher DNA concentration closer to the bottom. For sessile benthic organisms, DNA movement in the water column is likely to be restricted and sampling a few meters away from the source could impede DNA detection This should be especially true for invertebrates, as they are known to have lower shredding rates than fish (Andruzskiewicz Allan et al., 2021), which are also mobile organisms able to disperse their DNA signal as they move.

We detected very low DNA concentration with ddPCR across all our environmental and sampling parameters combination, except for a single sampling day in summer. Our findings are consistent with other studies using ddPCR to detect mollusk species (Mauvisseau et al., 2019), where alternative methods such as qPCR proved to be inefficient to detect the species (*Isogenus nubecula*) whereas ddPCR measured low DNA concentration (maximum 0.15 copies/μL).

The seasonal effect we obtained with higher DNA concentration in summer than in autumn seems to be driven by the exceptional concentration measured during a single summer day. When we control for this particular day by adding a dummy reproduction variable in the model, the seasonal effect is no longer significant. This highlights a weak or limited seasonal effect on detectability and DNA concentration (Figure 3). Other studies on macroinvertebrates in freshwater systems highlighted higher DNA concentration in warmer months, which authors attributed to higher activity, DNA shedding rates as well as reproductive events (Curtis et al., 2020; Wacker et al., 2019). A potential explanation for the overall low DNA concentration we measured could be a low DNA shedding rate from this species, which is known to filter 6 L/day when oysters and mussels can filter 40 and 100 L/day, respectively (Marin et al., 2019). As we detected an increased seasonal DNA concentration only on a particular day, we build on recent work to suggest how increased or peak eDNA concentration measured over time can suggest a reproductive event (Buxton et al., 2017; Ip et al., 2022; Takeuchi et al., 2019; Tsuji & Shibata, 2021). While we do not have the data to go beyond a putative claim, no other ecological parameter can realistically explain such a sharp increase in eDNA concentration over such a short time frame with similar environmental conditions.

Our results suggest the detection of a reproductive event during the summer, enhancing the level of DNA concentration significantly compared to the other summer day or autumn samples. Our interpretation remains hypothesis as we did not sample for any larvae or gametes, alternatively an eDNA‐focused analysis might prove useful to suggest a spawning event by measuring the nuclear versus mitochondrial DNA concentration ratio in samples. The relative quantity of nuclear DNA would increase during reproductive events due to the important presence of gametes compared to samples from non‐spawning events (Bylemans et al., 2017). In an environmental management context, it would be advisable to sample during reproductive season to increase detectability, but if gamete emissions are restricted to a short time frame (e.g., mass spawning over a few days only), they may be missed. We would rather suggest using the properties of eDNA to monitor a species' DNA concentration over time to detect the exact timing of reproduction events for understudied species. For *P. nobilis*, this information is of crucial importance considering its almost disappearance in marine seas, so any recolonization event would likely stem from parasite‐free brackish lagoons. Recent observations suggest recolonization might have started locally, with a small juvenile population of eight shells detected in Port‐Cros (coastal France) in August 2020 reported still alive in March 2021 (Ruitton & Lefebvre, 2021). Knowing the exact reproduction timing could allow us to model larval dispersal and predict a potential recolonization pattern into marine seas or identify the potential origin of newly settled juveniles (Kersting et al., 2020).

## AUTHOR CONTRIBUTIONS

**Virginie Marques:** Conceptualization (lead); data curation (lead); formal analysis (lead); investigation (lead); methodology (equal); writing – original draft (lead). **Simon Blanchet:** Conceptualization (equal); funding acquisition (equal); investigation (equal); methodology (equal); project administration (equal); validation (equal); writing – review and editing (equal). **Geraldine Loot:** Data curation (equal); formal analysis (equal); investigation (equal); methodology (equal); validation (equal); writing – review and editing (equal). **Claude Miaud:** Conceptualization (equal); methodology (equal); writing – review and editing (equal). **Serge Planes:** Resources (equal); writing – review and editing (equal). **Claire Peyran:** Methodology (equal); resources (equal); writing – review and editing (equal). **Veronique Arnal:** Methodology (equal); writing – review and editing (equal). **Coralie Calvet:** Conceptualization (equal); funding acquisition (equal); project administration (equal); writing – review and editing (equal). **Sylvain Pioch:** Conceptualization (equal); funding acquisition (equal); project administration (equal); writing – review and editing (equal). **Stephanie Manel:** Conceptualization (equal); formal analysis (equal); investigation (equal); methodology (equal); project administration (equal); resources (equal); supervision (equal); writing – original draft (supporting); writing – review and editing (equal).

## CONFLICT OF INTEREST STATEMENT

The authors declare no conflict of interest.

## Supporting information


Data S1.

File S1.

Table S2.

## Data Availability

All data are available in Dryad DOI: 10.5061/dryad.jh9w0vtj8, visible after manuscript publication. The repository contains the data and code. The data (csv file) were uploaded for review in the present submission.
