# Supplementary material for: Optimizing detectability of the endangered fan mussel using eDNA and ddPCR
Source: Ecol Evol. 2024 Jan 28;14(1):e10807. doi: 10.1002/ece3.10807 (PMC10822771; doi:10.1002/ece3.10807)
Supplement: Supplementary file 1 — Data S1. [file ECE3-14-e10807-s003.docx]

**Supl. Mat.**

**Table S1**. Primer and probe names, sequences, and parameters. Melting temperature was determined with the Geneious software.

| Primer name | Sequence (5’ – 3’) | GC content (%) | Tm (°C) | Amplicon size (bp) |
| --- | --- | --- | --- | --- |
| PN_COIM15-F | TCAGCTTTTGTAGAGGGCGG | 55 | 59 | ~202 |
| PN_COIM15-R | AGAGACTACCAACAGCACAGC | 52 | 58 |  |
| PN_COIM15-Probe | TGGATTTGTTCCCTTGGGCTGTTC | 50 | - | - |

**Table S2.** (submited as csv table). Localities and sampler of tissue samples used to test markers and the sequence obtained after amplification with the COI_M15F/R primers.

**Table. S3** GLM Model (poisson regression) outputs, without the dummy variable for the putative reproduction day.

|  | Estimate | Std.Error | zvalue | Pr(>\|z\|) |
| --- | --- | --- | --- | --- |
| (Intercept) | -2.39168 | 0.64517 | -3.707 | 0.00021*** |
| Depth | 0.15736 | 0.06443 | 2.442 | 0.01459* |
| SiteCanal_Sete | -0.26049 | 0.18962 | -1.374 | 0.16953 |
| seasonsummer | 3.77779 | 0.63584 | 5.941 | 2.83E-09*** |

**Table S4.** Additional candidate primers designed during marker development.

| Marker name | Primer forward | Primer reverse | Amplicon size (bp) |
| --- | --- | --- | --- |
| PN_COIM18 | CAGGGTTTTTGGGGGATGGG | TAGCCAGTTTCCAAACCCCC | 92 |
| PN_16SM8 | TAACTTTCGCGTGCAAAGGC | TGTCAAGCCCGCTAAAGAGC | 160 |


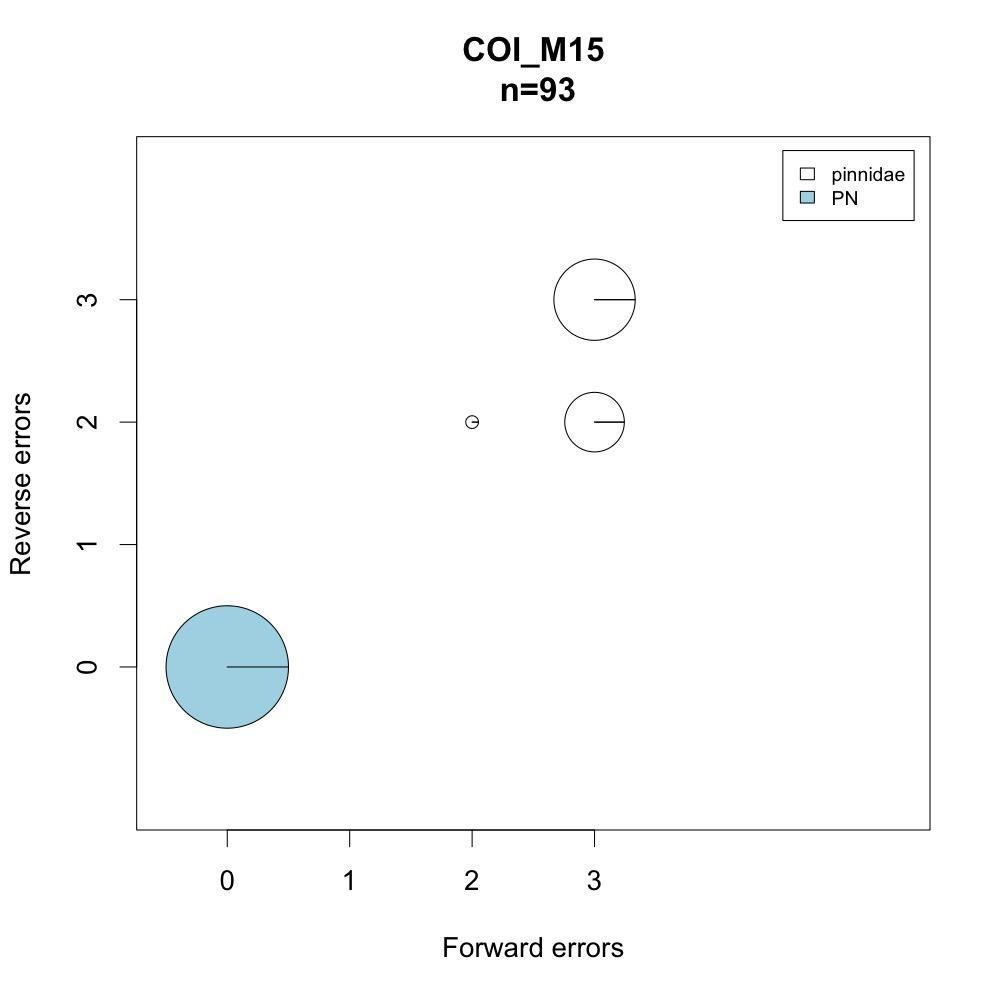


**Figure S1**. Number of mismatches on forward and reverse primers of the COI_M15 marker developed to target *Pinna nobilis* and their amplifications. Blue color represents *P. nobilis* sequences and white color represents sequences from other Pinnidae absent from the Mediterranean region.


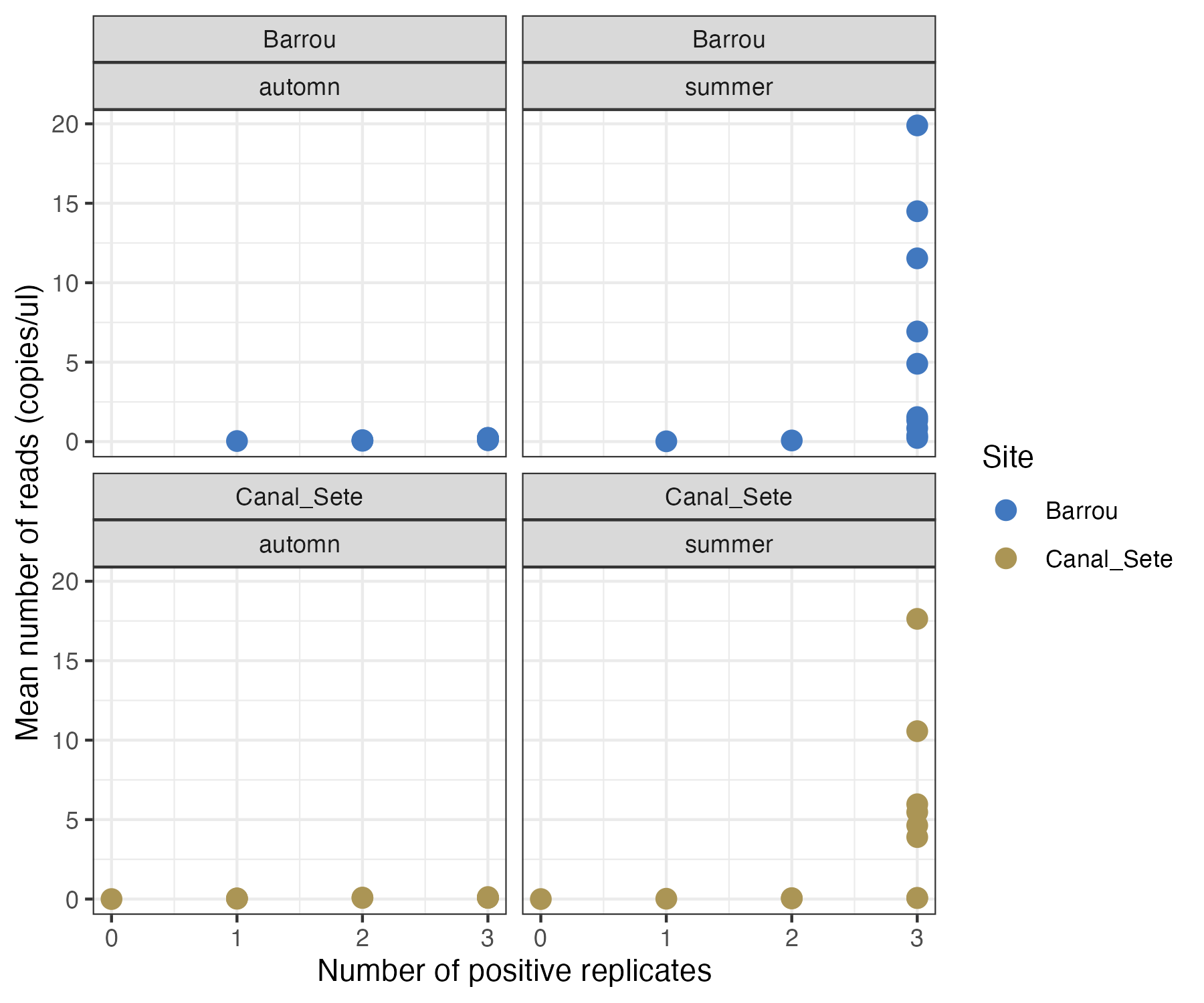


**Figure. S2.** A comparison of the number of positive replicates with ddPCR considering the number of copies measured and the site and season. Blue and gold dots represent ddPCR results for each site.

**Figure. S3.** GLM effects of parameters without considering any putative reproduction event.
